# Notice of correction: Acta Orthopaedica 81(2)

**DOI:** 10.3109/17453674.2010.526042

**Published:** 2010-10-08

The online issue of Acta Orthopaedica 81(2) initially published on 8 April 2010 contained an error in the order of contents. The article by Kuribayashi et al. “Vitamin E prevents steroid-induced osteonecrosis in rabbits”, previously published in 81(1), was incorrectly entered in place of Kock et al. “Bone scintigraphy after osteochondral autograft transplantation in the knee” which had appeared in the print issue.

To correct this mistake the running order of the online issue was amended to match the print version on 29 June 2010.

As a result of our error, citations for Acta Orthopaedica 81(2) in the National Library of Medicine's Medline Index are not correct.

The correct citation for:

Kuribayashi M, Fujioka M, Takahashi KA, Arai Y, Ishida M, Goto T, Kubo T. Vitamin E prevents steroid-induced osteonecrosis in rabbits. Acta Orthop. 2010 Apr; 81(2): 206-12. (PubMed PMID: 20146637; PubMed Central PMCID: PMC2852158)

Should be:

Kuribayashi M, Fujioka M, Takahashi KA, Arai Y, Ishida M, Goto T, Kubo T. Vitamin E prevents steroid-induced osteonecrosis in rabbits. Acta Orthop. 2010 Feb; 81(1): 154-60. (PubMed PMID: 20170436; PubMed Central PMCID: PMC2856221).

The article by Kock et al. is currently not in the index, but should appear as:

Kock NB, van Tankeren E, Oyen WJG, Wymenga AB, van Susante JLC. Bone scintigraphy after osteochondral autograft transplantation in the knee. Acta Orthop. 2010 Feb; 81(1): 206-10.

In addition, due to the difference in the number of pages between Kuribayashi et al. and Kock et al., all subsequent papers in this issue are indexed with incorrect page numbers on Medline.

Raiss P, Pape G, Jäger S, Loew M, Bitsch R, Rickert M. In vitro measurement of temperature changes during implantation of cemented glenoid components. Acta Orthop. 2010 Apr; 81(2): 213-7. PubMed PMID: 20367412; PubMed Central PMCID: PMC2895340.

The correct citation for Raiss et al. is: Acta Orthop. 2010 Apr; 81(2): 211-5.

Heineman DJ, Poolman RW, Nork Sean SE, Ponsen KJ, Bhandari M. Plate fixation or intramedullary fixation of humeral shaft fractures. Acta Orthop. 2010 Apr; 81(2): 218-25. Review. PubMed PMID: 20170424; PubMed Central PMCID: PMC2895341.

The correct citation for Heineman et al. is: Acta Orthop. 2010 Apr; 81(2): 216-23.

Karlsson MK, Herbertsson P, Nordqvist A, Besjakov J, Josefsson PO, Hasserius R. Comminuted fractures of the radial head. Acta Orthop. 2010 Apr; 81(2): 226-9. PubMed PMID: 20367419; PubMed Central PMCID: PMC2895342.

The correct citation for Karlsson et al. is: Acta Orthop. 2010 Apr; 81(2): 224-7.

Giannicola G, Sacchetti FM, Greco A, Gregori G, Postacchini F. Open reduction and internal fixation combined with hinged elbow fixator in capitellum and trochlea fractures. Acta Orthop. 2010 Apr; 81(2): 230-5. PubMed PMID: 20180722; PubMed Central PMCID: PMC2895343.

The correct citation for Giannicola et al. is: Acta Orthop. 2010 Apr; 81(2): 228-33.

Aspenberg P, Johansson T. Teriparatide improves early callus formation in distal radial fractures. Acta Orthop. 2010 Apr; 81(2): 236-8. PubMed PMID: 20367417; PubMed Central PMCID: PMC2895344.

The correct citation for Aspenberg et al. is: Acta Orthop. 2010 Apr; 81(2): 234-6.

Nilsson A, Wiig M, Alnehill H, Berggren M, Björnum S, Geijer M, Kopylov P, Sollerman C. The Artelon CMC spacer compared with tendon interposition arthroplasty. Acta Orthop. 2010 Apr; 81(2): 239-46. PubMed PMID: 20180717; PubMed Central PMCID: PMC2895345.

The correct citation for Nilsson et al. is: Acta Orthop. 2010 Apr; 81(2): 237-44.

Lofterød B, Terjesen T. Changes in lower limb rotation after soft tissue surgery in spastic diplegia. Acta Orthop. 2010 Apr; 81(2): 247-51. PubMed PMID: 20175660; PubMed Central PMCID: PMC2895346.

The correct citation for Lofterød et al. is: Acta Orthop. 2010 Apr; 81(2): 245-9.

Paul L, Docquier PL, Cartiaux O, Cornu O, Delloye C, Banse X. Selection of massive bone allografts using shape-matching 3-dimensional registration. Acta Orthop. 2010 Apr; 81(2): 252-7. PubMed PMID: 20175643; PubMed Central PMCID: PMC2895347.

The correct citation for Paul et al. is: Acta Orthop. 2010 Apr; 81(2): 250–5

Slobogean GP, O'Brien PJ, Brauer CA. Single-dose versus multiple-dose antibiotic prophylaxis for the surgical treatment of closed fractures. Acta Orthop. 2010 Apr; 81(2): 258-64. PubMed PMID: 20148647; PubMed Central PMCID: PMC2895348.

The correct citation for Slobogean et al. is: Acta Orthop. 2010 Apr; 81(2): 256-62.

Men X, Han S, Gao J, Cao G, Zhang L, Yu H, Lu H, Pu J. Taurine protects against lung damage following limb ischemia reperfusion in the rat by attenuating endoplasmic reticulum stress-induced apoptosis. Acta Orthop. 2010 Apr; 81(2): 265-9. PubMed PMID: 20148646; PubMed Central PMCID: PMC2895349.

The correct citation for Men et al. is: Acta Orthop. 2010 Apr; 81(2): 263-7

Dumont CE, Schuster AJ, Freslier-Bossa M. Borggreve-Van Nes rotationplasty for infected knee arthroplasty - a case report. Acta Orthop. 2010 Apr; 81(2): 270-2. PubMed PMID: 20170417; PubMed Central PMCID: PMC2895350.

The correct citation for Dumont et al. is: Acta Orthop. 2010 Apr; 81(2): 268-70.

The publishers apologize to the relevant authors and our readers for these errors. We trust that, following publication of this notice, the National Library of Medicine will be willing to correct the Medline index.

